# Detection and Occurrence of Plasmid-Mediated AmpC in Highly Resistant Gram-Negative Rods

**DOI:** 10.1371/journal.pone.0091396

**Published:** 2014-03-18

**Authors:** E. Ascelijn Reuland, John P. Hays, Denise M. C. de Jongh, Eman Abdelrehim, Ina Willemsen, Jan A. J. W. Kluytmans, Paul H. M. Savelkoul, Christina M. J. E. Vandenbroucke-Grauls, Nashwan al Naiemi

**Affiliations:** 1 Medical Microbiology and Infection Control, VU University Medical Center, Amsterdam, The Netherlands; 2 Department of Medical Microbiology and Infectious Diseases, Erasmus MC, Rotterdam, The Netherlands; 3 Department of Medical Microbiology and Infection Control, Amphia Hospital, Breda, The Netherlands; 4 Laboratory for Medical Microbiology and Public Health, Hengelo, The Netherlands; 5 Medical Microbiology and Infection Control, Ziekenhuisgroep Twente, Almelo, The Netherlands; Aligarh Muslim University, India

## Abstract

**Objectives:**

The aim of this study was to compare the current screening methods and to evaluate confirmation tests for phenotypic plasmidal AmpC (pAmpC) detection.

**Methods:**

For this evaluation we used 503 Enterobacteriaceae from 18 Dutch hospitals and 21 isolates previously confirmed to be pAmpC positive. All isolates were divided into three groups: isolates with 1) reduced susceptibility to ceftazidime and/or cefotaxime; 2) reduced susceptibility to cefoxitin; 3) reduced susceptibility to ceftazidime and/or cefotaxime combined with reduced susceptibility to cefoxitin. Two disk-based tests, with cloxacillin or boronic acid as inhibitor, and Etest with cefotetan-cefotetan/cloxacillin were used for phenotypic AmpC confirmation. Finally, presence of pAmpC genes was tested by multiplex and singleplex PCR.

**Results:**

We identified 13 pAmpC producing Enterobacteriaceae isolates among the 503 isolates (2.6%): 9 CMY-2, 3 DHA-1 and 1 ACC-1 type in *E. coli* isolates. The sensitivity and specificity of reduced susceptibility to ceftazidime and/or cefotaxime in combination with cefoxitin was 97% (33/34) and 90% (289/322) respectively. The disk-based test with cloxacillin showed the best performance as phenotypic confirmation method for AmpC production.

**Conclusions:**

For routine phenotypic detection of pAmpC the screening for reduced susceptibility to third generation cephalosporins combined with reduced susceptibility to cefoxitin is recommended. Confirmation via a combination disk diffusion test using cloxacillin is the best phenotypic option. The prevalence found is worrisome, since, due to their plasmidal location, pAmpC genes may spread further and increase in prevalence.

## Introduction

The frequency of highly resistant gram-negative rods (HR-GNRs) is still increasing worldwide [1]. Gram-negative rods with resistance to carbapenems or to third generation cephalosporins only due to ESBL-production were defined as highly resistant isolates. Furthermore, strains resistant to two agents of the antimicrobial groups quinolones and aminoglycosides were also defined as highly resistant (adapted from the Dutch guideline for preventing nosocomial transmission of highly resistant microorganisms (HRMO)) [2].

Apart from ESBLs, one class of these enzymes has received relatively little attention, namely the AmpC-type beta-lactamases. Although these “Class C” beta-lactamases are often found to be associated with the bacterial chromosome, an increasing prevalence of plasmid-encoded AmpC enzymes (pAmpC) has been reported [3]–[5]. Traditionally, chromosomally encoded AmpC is mainly present in group II Enterobacteriaceae (*Enterobacter* spp., *Citrobacter freundii*, *Hafnia alvei*, *Providencia* spp., *Serratia* spp., *Morganella morganii*), but pAmpC is gaining more and more importance in group I Enterobacteriaceae (*Proteus mirabilis*, *Klebsiella* spp., *Salmonella* spp., *Escherichia coli*, and *Shigella* spp.) [3]. Furthermore, carriage of plasmid-mediated AmpC is often associated with multidrug resistance (e.g. resistance to aminoglycosides, quinolones and cotrimoxazole), and worryingly, isolates with porin loss that carry pAmpC may also be resistant to carbapenems [4], [6], [7]. The occurrence of pAmpC has been investigated in several studies [6], [8]–[10]. In a selection of clinical Enterobacteriaceae from a national survey a high prevalence of ampC genes among Enterobacteriaceae was found; 32 out of 181 isolates with reduced susceptibility to cefoxitin concerned pAmpC [11]. Another study showed a high prevalence of ESBL/AmpC-producing *E. coli* in birds and farmers at Dutch broiler farms [12].

The prevalence of pAmpC carriage reported in these studies is still low, though this is most likely an underestimation due to the difficulties associated with routine phenotypic screening for pAmpC. This means that molecular detection techniques are the current ‘gold standard’ for the detection of pAmpC, although these are more expensive and difficult to implement for routine use [3], [13]. For this reason, several previous studies have attempted to compare and evaluate current phenotypic tests for the detection of pAmpC [14]–[16]. However, most of these reports did not analyze different screening methodologies. Therefore, the objective of this study was to compare the current pAmpC phenotypic screening methodologies used in the literature and to evaluate the different confirmation methods. The methodology was further used to assess the prevalence of pAmpC among 502 group I HR-GNRs collected from 18 Dutch hospitals in 2007.

## Materials and Methods

### Bacterial isolates

Bacterial isolates were retrospectively screened using a collection of group I HR-GNR Enterobacteriaceae previously collected during a prospective observational multicenter study in 18 hospitals in the Netherlands [17]. Gram negative rods were defined as highly resistant (HR-GNR), according to the criteria of the Dutch Working Party on Infection Prevention [2]. Isolates were obtained from patients hospitalized between January 1 and October 1, 2007 and comprised strains isolated from clinical and screening specimens. In total 892 different HR-GNR isolates were recovered from 786 patients.

Identification of strains, susceptibility testing and ESBL detection was performed according to Dutch guidelines [17], [18]. ESBL-encoding genes (*bla*_CTX-M_, *bla*_SHV_ and *bla*_TEM_), *bla*_OXA_ and carbapenemase-encoding genes (*bla*_KPC_, *bla*_NDM_, *bla*_OXA-48_, *bla*_IMP_ and *bla*_VIM_) were detected by microarray and if necessary confirmed by PCR and sequencing (BaseClear) at the VU University Medical Center (VUmc) [19], [20]. The authors specifically focused on Enterobacterial species that are known to lack a chromosomal AmpC gene (*P. mirabilis*, *Klebsiella* spp., *Salmonella* spp.), or that are known to carry a chromosomal AmpC gene, but produce only low levels of AmpC enzyme (*E. coli* and *Shigella* spp.). Therefore, 503 of the 892 HR-GNR isolates from the original study were included in the present study. The 503 highly resistant isolates comprised *E. coli* (333), *Klebsiella* spp. (123), *Proteus* spp. (42), *Salmonella* spp. (3) and *Shigella* spp. (2). Duplicate isolates from the same patient were excluded; isolates were obtained from screening samples (158), and from clinical samples (345); in 61 samples the HRMO was also found in blood cultures during hospitalization. The samples were obtained from 18 different hospitals. Finally, a further 21 pAmpC-producing isolates, previously characterized by PCR, were included (as positive controls) in the study collection. Fifteen of the pAmpC control strains were obtained from the isolate biobank available at Erasmus Medical Center, having been collected from various non-Dutch sources over different years. The isolates were identified as *E. coli* by classical biochemical methods and confirmed to be pAmpC positive by PCR. Six isolates (confirmed by PCR at the Erasmus MC) were isolated during a study on community-acquired ESBL-producing Enterobacteriaceae at the VU University Medical Center, between August 12 and December 13, 2011.

### Screening for AmpC

Three screening strategies were evaluated: reduced susceptibility to third generation cephalosporins, reduced susceptibility to cefoxitin, and a combination of reduced susceptibility to third generation cephalosporins and cefoxitin [21]. Reduced susceptibility to third generation cephalosporins was defined as a MIC for cefotaxime and/or ceftazidime that was >1 mg/L, corresponding to inhibition zone diameters for cefotaxime of ≤27 mm and for ceftazidime of ≤22 mm (following ESBL screening protocols defined by Dutch national guidelines) [18]. Reduced susceptibility to cefoxitin was determined using Vitek 2 (bioMerieux, Marcy-l'Etoile, France), and was defined as a MIC>8 mg/L according to EUCAST guidelines [21]. If isolates were positive for pAmpC by PCR but susceptible to cefoxitin (MIC≤8 mg/L; inhibition zone >18 mm), Vitek testing was repeated and phenotypic testing using cefoxitin Etest (bioMérieux, Solna, Sweden) on Mueller Hinton agar was performed to ensure cefoxitin sensitivity.

### Confirmation of AmpC

AmpC production was confirmed phenotypically using a two disk-based test and an Etest with boronic acid or cloxacillin as inhibitors. The combination disk diffusion tests consisted of cefotaxime and ceftazidime combined with boronic acid or cloxacillin as inhibitor (Rosco, Taastrup, Denmark). A positive test was considered when the zone of inhibition was ≥5 mm larger than the zone generated without inhibitor. The Etest cefotetan/cefotetan-cloxacillin (CN/CNI, bioMérieux, Marcy-l'Etoile, France) methodology was also used to confirm AmpC production, where either a ratio of cefotetan/cefotetan-cloxacillin ≥8, deformation of the ellipse, or the presence of a phantom zone were interpreted as positive for an AmpC producer.

### Molecular pAmpC gene screening

Isolates that were suspected to be pAmpC producers by one or more of the screening methods were further tested by multiplex PCR. Thus, all 335 isolates with reduced susceptibility to third generation cephalosporins and/or reduced susceptibility to cefoxitin, were analyzed by PCR. DNA was isolated using the easyMag system (bioMerieux, Marcy-l'Etoile, France). Plasmid-mediated AmpC types were characterized using a variation of a standard multiplex PCR (Erasmus MC, Rotterdam) that can identify six family-specific pAmpC genes: *bla*_CMY II_, *bla*_MOX_, *bla*_FOX_, *bla*_DHA_, *bla*_ACT/MIR_ and *bla*_ACC_ genes [13]. In this variation, the annealing temperature was increased to 70°C and multiplex PCR positive isolates were further tested using specific singleplex AmpC PCRs under the same reaction conditions, to ensure that the PCR-products found in the multiplex PCR positive isolates were correct. This multiplex AmpC PCR methodology was used as the gold standard AmpC detection methodology.

Analysis of genetic relatedness among the tested isolates in this study was performed using amplified-fragment length polymorphism (AFLP) as described by Savelkoul *et al.* [22] Clustering and interpretation of AFLP banding patterns were performed using BioNumerics software, version 6.6 (Applied Maths, Sint-Martens-Latem, Belgium).

### Data analysis

Statistical analyses were performed using SPSS, version 20.0. Sensitivity and specificity of the screening and confirmation methods were calculated using multiplex AmpC PCR results as the gold standard.

## Results

### Phenotypic detection methodologies for (plasmid-mediated) AmpC

Of the 503 HR-GNR isolates from Dutch hospitals 335 isolates (67%) showed reduced susceptibility to third generation cephalosporins and/or reduced susceptibility to cefoxitin. In addition three isolates had increased MICs (>0.25 mg/L) for meropenem (2 mg/L, 4 mg/L and 8 mg/L) and imipenem (4 mg/L, 2 mg/L and 4 mg/L, respectively) [23]. The number of isolates for each species included *E. coli* (224), *Klebsiella* spp. (106), *Proteus* spp. (4) and *Salmonella* spp. (1). In total 101 screenings samples were isolated, the remaining 234 samples were from clinical samples. Of these, 12.9% (43/335) isolates were detected in blood cultures at a later stage. Nearly half of the samples (42.4%, 142/335) were obtained on the Intensive Care Unit.

Thirteen out of these 335 (3.9%) isolates were found to be pAmpC positive using a multiplex pAmpC PCR, i.e. CMY-2 (9), DHA-1 (3) and ACC-1 (1). Also included in the phenotypic screening was a collection of 21 previously characterized pAmpC positive *E. coli* isolates, 20 CMY-2 and one isolate with DHA-1 (data not published), generating a total of 356 isolates for phenotypic comparison and evaluation (Table 1). Using both screening and confirmatory phenotypic methodologies on these 356 isolates revealed three major phenotypic groups. Phenotypic group I comprised 327 isolates that were found to be reduced susceptible to third generation cephalosporins (regardless of resistance to cefoxitin), with 34 (10.4%) of these isolates being pAmpC positive by PCR. This group included all pAmpC PCR-positive isolates. This results in a sensitivity of 100% (34/34) and a specificity of 9% (29/322).

**Table 1:** AmpC production in 356 highly resistant enterobacterial isolates.

Species	Total collection	pAmpC positive	pAmpC type
Total	356	34	
*Escherichia coli*	245 (68.8%)	28 (82.4%)	27 CMY-2, 1 DHA-1
*Klebsiella pneumoniae*	82 (23.0%)	4 (11.8%)	3 DHA-1, 1 CMY-2
*Klebsiella oxytoca*	24 (6.8%)	1 (2.9%)	ACC-1
*Proteus mirabilis*	4 (1.1%)	1 (2.9%)	CMY-2
*Salmonella species*	1 (0.3%)	0	

*The 356 isolates were selected based on resistance to third generation cephalosporins (cefotaxime and/or ceftazidime) and/or cefoxitin. Reduced susceptibility was defined as a MIC>1 mg/L corresponding to inhibition zone diameter of ≤27 mm for cefotaxim, MIC>1 mg/L corresponding to inhibition zone diameter of ≤22 mm for ceftazidim, MIC>8 mg/L for cefoxitin.*

Phenotypic group II comprised 122 isolates with reduced susceptibility to cefoxitin (regardless of reduced susceptibility to third generation cephalosporins). Thirty three of these 122 (27%) isolates were found to be pAmpC positive by PCR. An ACC-1 gene positive isolate remained undetected due to a lack of cefoxitin resistance. This results in a sensitivity of 97% (33/34) and a specificity of 72% (233/322).

Phenotypic group III comprised 66 isolates with reduced susceptibility to cefoxitin combined with reduced susceptibility to third generation cephalosporins. Thirty three of these 66 (50%) isolates were found to be pAmpC positive by PCR, but again the ACC-1-positive isolate remained undetected. These results generated a sensitivity of 97% (33/34), but a higher specificity of 90% (289/322).

The performance of different AmpC confirmatory tests in combination with different antibiotic and inhibitor combinations is shown in Table 2. A maximum sensitivity of 94% (range 91%–94%) was obtained for all the three screening strategies in combination with the combination disk diffusion tests with cloxacillin. By combining reduced susceptibility to third generation cephalosporins with reduced susceptibility to cefoxitin and in combination with the inhibitor-based combination disk diffusion test using cloxacillin yielded a sensitivity of 91% (31/34) with a specificity of 96% (309/322).

**Table 2:** Comparison of phenotypic pAmpC confirmation tests.

Phenotypic detection methods*	Total Number of isolates positive by pAmpC PCR	Total Number of isolates pAmpC positive using phenotypic methods	Sensitivity (%)	Specificity (%)
***Phenotypic Group I:***				
***Analysis after screening for reduced susceptibility to third generation cephalosporins (n = 327)***	***34***	***34***	***100***	***9***
DDCT with cloxacillin		32	94	56
DDCT with boronic acid		30	88	65
Etest CN/CNI		27	79	98
***Phenotypic Group II:***				
***Analysis after screening for reduced susceptibility to cefoxitin (n = 122)*** **	***34***	***33*** **	***97***	***72***
DDCT with cloxacillin		31	91	93
DDCT with boronic acid		29	85	92
Etest CN/CNI		27	79	98
***Phenotypic Group III:***				
***Analysis after screening for reduced susceptibility to all these cephalosporins together (n = 66)*** **	***34***	***33*** **	***97***	***90***
DDCT with cloxacillin		31	91	96
DDCT with boronic acid		29	85	95
Etest CN/CNI		27	79	98

*The antibiotics used in the DDCT tests were cefotaxime and ceftazidime combined with cloxacilllin or boronic acid. Etest CN/CNI consisted of cefotetan (CN) with cefotetan-cloxacillin (CNI).*

*DDCT with cloxacillin/boronic acid and CN/CNI Etest.

**Sensitivity and specificity of these confirmation tests is performed without ACC-1 in the analysis due to susceptibility for cefoxitin.

The DDCT with boronic acid missed two *E. coli* with CMY-2. The CN/CNI Etest did not detect three *E. coli* with CMY-2, one *E. coli* with DHA-1 and one *Klebsiella oxytoca* with ACC-1. Importantly, two *E. coli* isolates possessing CMY-2 type enzymes, one coproducing CTX-M-1 and one coproducing OXA-1, were not detected with any of the confirmation methodologies shown in Table 2. No other isolates additionally producing ESBL were negative in the phenotypic confirmation. All isolates with partly negative confirmation results were fully resistant to cefotaxime, ceftazidime and/or cefoxitin.

### Molecular epidemiology

Multiplex pAmpC PCR screening revealed a prevalence of 2.6% (13/503) for pAmpC carriage among the group I Enterobacteriaceae tested in this study. Further molecular analysis revealed that nine of the 13 pAmpC multiplex PCR-positive isolates, obtained in the multicenter study and isolated in five of the 18 different hospitals between between January 1 and October 1, 2007, contained the CMY-2 gene (predominantly *E. coli* except for one *P. mirabilis* and one *Klebsiella pneumoniae*). Three isolates contained DHA-1 (all *K. pneumoniae*) and one isolate ACC-1 (*K. oxytoca*). Two of these isolates, one *E. coli* with CMY-2 and one *K. pneumoniae* with DHA-1, were obtained out of screening material and the rest were clinical samples.

In three of these 13 pAmpC isolates an ESBL-encoding gene was also detected, these ESBL genes were determined as CTX-M-1 group (2/13) and CTX-M-9 group (1/13).

The 15 pAmpC-producers from the isolate biobank available at Erasmus Medical Center (Erasmus MC) were obtained from various non-Dutch sources over different years, therefore no identical strains were expected. However, AFLP was performed on the 6 pAmpC isolates derived from the community-acquired ESBL-producing strains to assure that these isolates were not identical. Furthermore, AFLP analysis of the 13 pAmpC producers from the multicentre study revealed no epidemiological relationship.

## Discussion

Our results show that among HR-GNR in Dutch hospitals, 2.6% (13/505) pAmpC-producing isolates were found retrospectively in a selected subgroup of group I Enterobacteriaceae. That the majority of isolates possessed CMY-2 type pAmpC is in line with molecular epidemiological results published elsewhere [6], [11], [24], [25].

Several reports reveal that pAmpC-producing nosocomial isolates have become endemic in some hospitals that they can cause outbreaks, and that they affect therapeutic choices [26]–[28]. Reports from Spain suggest that compared to ESBL-producing organisms the acquisition of pAmpC-producing Enterobacteriaceae is still mainly hospital- or healthcare-associated [6]. The isolates in our study were clinical isolates, but we cannot differentiate between healthcare-associated or community-based sources of nosocomial pAmpC infections. A rise in pAmpC carriage and infections could in theory mirror the rapid increase in global Enterobacterial ESBL isolates observed over the last 10 years, not least because pAmpC carriage is reportedly becoming a serious global infectious disease health concern [5].

Of great concern, treatment of infections caused by pAmpC-producing strains with cephalosporins is associated with adverse clinical outcomes [29], [30].

Recently Gude *et al.* evaluated different AmpC confirmatory tests [14]. In contrast to these authors, we found not the Etest but DDCT cloxacillin as the best test, with the best sensitivity and specificity after the combination of screening criteria. In general the same genes were identified, except that we detected also *bla*_ACC-1_. This difference may be due to differences in the selection of strains. We included not only cefoxitin-resistant strains, but also strains of group I Enterobacteriaceae that were resistant to third generation cephalosporins alone. Therefore, cefoxitin susceptible isolates producing pAmpC (ACC-1) could be detected. In addition a MIC>8 mg/L was used as breakpoint for cefoxitin, as to eliminate less resistant isolates. These more stringent MICs were used to detect more isolates that fulfilled the screening criteria. We used the same cefotetan/cefotetan-cloxacillin Etest, however the other phenotypic tests were DDCT with cefotaxime/ceftazidime combined with boronic acid or cloxacillin as inhibitor. The latter were selected because these are commercially available, cheap and less prone to interobserver variability (like for example a three dimensional (3D) test or double disk approximation test).

The use of molecular testing strategies such as multiplex AmpC PCRs are currently the gold standard for pAmpc detection. A more convenient strategy for many institutions would be to optimize the phenotypic screening and confirmatory methodologies that are currently available in order to maximize the sensitivity and specificity of pAmpC detection. Results using pAmpC phenotypic screening assays on our set of isolates showed that a reduced susceptibility to cefotaxime and/or ceftazidime alone generated the best sensitivity (100%, i.e. 34/34). However, a major disadvantage of this methodology was found to be a low specificity (9%, i.e. 29/322) of detection. This means that no false negative results were generated using this methodology, but that there was a relatively high frequency of false positives. The end result is that many unnecessary confirmatory tests would have to be performed using this methodology.

In general resistance to cefoxitin is often used as indicator for the production of class C beta-lactamases (which include pAmpC beta-lactamases), with most reports only investigating isolates resistant to cephamycins [24], [31]. Though cefoxitin resistance is a sensitive test, it is not specific, mainly because a reduced permeability of the bacterial outer membrane, as well as the expression of some carbapenemase enzymes, may also lead to cefoxitin resistance [32], [33]. Further, hyperproduction of chromosomal AmpC, may lead to cephamycin resistance [3], [14], [34]. Another disadvantage of using cefoxitin resistance as a phenotypic screening methodology is that ACC-1-type enzymes are susceptible to cefoxitin, which means that isolates possessing these genes will be regarded as pAmpC negative. This is an important point, because ACC-type enzymes have been detected in several different countries in Europe, including a large outbreak in a teaching hospital in Garches, France [25], [27], [35].

From our results, we conclude that a combination of reduced sensitivity to the third generation cephalosporins (cefotaxime and/or ceftazidime) and reduced susceptibility to cefoxitin may generate the best specificity (90%) for phenotypic pAmpC screening (Table 2). A limitation however is that ACC-like enzymes will not be detected. In combination with this screening strategy our results suggest that the combination disk diffusion test with cloxacillin is the best phenotypic confirmation method.

With respect to the disk-based phenotypic confirmatory AmpC methodologies used, it is well known that boronic acid and cloxacillin are well-known inhibitors of AmpC [14], [16], [36]–[39]. Boronic acid is an AmpC inhibitor (both plasmidal and chromosomal) and also an inhibitor of KPC beta-lactamases. We found one isolate with a positive AmpC confirmation test with boronic acid but negative results using the test with cloxacillin. This *K. pneumoniae* isolate showed an increased MIC for meropenem (2 mg/L) and was KPC positive. Of the three isolates with MIC meropenem >0.25 mg/L one isolate (*K. pneumoniae*) was KPC positive. The two other isolates (one *K. pneumoniae* and one *E. coli*) were resistant to third generation cephalosporins and cefoxitin, showed decreased susceptibility to ertapenem, had negative AmpC confirmation test results and negative PCR result for KPC. Therefore, other mechanisms could be responsible for the resistance, e.g. porin loss and ESBLs (both isolates harboured SHV-type ESBL and CTX-M-1, respectively) or other carbapenemases.

In conclusion, our data suggest that phenotypic AmpC detection methods can be improved by combining the screening results of susceptibility testing to third generation cephalosporins and the susceptibility results to cefoxitin. Reduced susceptibility to both being a good indicator for the presence of pAmpC gene expression. However, it should be noted that the presence of ACC-1 type AmpC will still be missed using these combined methodologies. The presence of pAmpC can be confirmed with the combination disk diffusion test; cefotaxime and ceftazidime with cloxacillin showed the best results. For the future, it is desirable to evaluate a larger collection of different enterobacterial species with pAmpC and to perform more studies to define the frequency of occurrence of pAmpC in comparison to 2007.
